# Prevention of Bleomycin-Induced Pulmonary Inflammation and Fibrosis in Mice by Bilobalide

**DOI:** 10.1155/2023/1973163

**Published:** 2023-01-24

**Authors:** Xingcai Zhang, Wei Zhang, Xianhai Chen, Yuli Cai

**Affiliations:** ^1^Department of Respiratory and Critical Care Medicine, The Affiliated Hospital of Shandong University of Traditional Chinese Medicine, Jinan, Shandong 250011, China; ^2^Department of Joint Orthopedics, The Affiliated Hospital of Shandong University of Traditional Chinese Medicine, Jinan, Shandong 250011, China

## Abstract

Idiopathic pulmonary fibrosis (IPF) is a fatal interstitial lung disease. Bilobalide (BB) is a sesquiterpene isolated from *Ginkgo biloba*, and its role in IPF is poorly understood. Mice were intratracheally instilled with 2.5 mg/kg bleomycin (BLM) to induce IPF and then treated with 2.5, 5, and 10 mg/kg BB daily for 21 days. Treatment with BB ameliorated pathological injury and fibrosis of lung tissues in BLM-induced mice. BB suppressed BLM-induced inflammatory response in mice as demonstrated by reduced inflammatory cells counts (leukocytes, neutrophils, macrophages, and lymphocytes) and pro-inflammatory factors (CCL2 and TNF-*α*), as well as increased CXCL10 levels in BALF. The expression of BLM-induced hydroxyproline, LDH, and pro-fibrotic mediators including fibronectin, collagen I, *α*-smooth muscle actin (*α*-SMA), transforming growth factor (TGF)-*β*1, matrix metalloproteinase (MMP)-2, and MMP-9 in lung tissue was inhibited by BB treatment, and the tissue inhibitor of metalloproteinase-1 (TIMP-1) expression was increased. BB blocked the phosphorylation of JNK and NF-*κ*B, and the nuclear translocation of NF-*κ*B in the lung tissue of mice induced by BLM. Additionally, it abated the activation of NLRP3 inflammasome in lung tissue induced by BLM, which led to the downregulation of IL-18 and IL-1*β* in BALF. Our present study suggested that BB might ameliorate BLM-induced pulmonary fibrosis by inhibiting the early inflammatory response, which is probably via the inhibition of the JNK/NF-*κ*B/NLRP3 signal pathway. Thus, BB might serve as a therapeutic potential agent for pulmonary inflammation and fibrosis.

## 1. Introduction

Idiopathic pulmonary fibrosis (IPF) is a common chronic progressive fatal lung disease characterized by alveolar epithelial cell damage, chronic inflammation, and abnormal deposition of extracellular matrix (ECM), leading to progressive decline in lung function [1, 2]. It was reported that there were 20 new cases per 100,000 people per year [3], and it usually has a short median survival of about 3–5 years from the time of diagnosis [4]. Although IPF affects millions of patients worldwide and causes scarring of the lungs, its etiology remains unclear [5]. In the past few years, several factors have been suggested to be involved in the pathogenesis of IPF, including inflammation, epithelial-mesenchymal transition (EMT), imbalance of ECM degradation and collagen deposition, and oxidative stress [6]. Oxidative stress and inflammatory response may be two important factors that trigger the disease and allow its progression [7, 8]. At present, treatment options for IPF are quite limited. Clinically, pirfenidone and nintedanib are the first-line agents for the treatment of mild and/or moderate IPF, but their prognostic results are not satisfactory [9, 10]. Thus, exploring effective antioxidant or anti-inflammatory drugs is of great significance for the treatment of IPF.

*Ginkgo biloba* is an ancient Chinese tree that has been used to treat a variety of ailments. Studies have shown that it has beneficial effects on a variety of pathological conditions, including hepatoprotection, photoprotective effects, DNA repair mechanisms, antioxidant, and anti-inflammatory activities [11]. Standardized ginkgo extract EGb761 is a well-known Ginkgo biloba extract that dates back over a century. It contains two main groups of bioactive components: 24% flavonol glycosides and 6% terpene trilactones [12]. Bilobalide (BB) is one of the main components of terpene trilactone [13], approximately accounting for 2.9–3.2% of EGb76 [14]. Currently, BB has been used as a phytopharmaceutical or food supplement in numerous countries [15]. Considerable evidence has been suggested that BB has a wide range of therapeutic applications for neurological and vascular injuries [16]. BB could activate Akt/Nrf2 signal pathway to reduce oxidative stress, thereby alleviating cerebral ischemia injury [17]. Additionally, BB has been proven to have an anti-inflammation effect. Goldie and Dolan [18] reported that BB could alleviate carrageenan-induced inflammation and inflammatory pain in rats by inhibiting inflammatory factor release. BB also inhibited the expression of interleukin (IL)-17-induced inflammatory factors and matrix metalloproteinases (MMPs) and alleviated inflammatory damage in ATDC5 cells [19]. However, an extensive literature and the patent search revealed a lack of data on the effects of BB on IPF.

Based on the inflammatory characteristics of IPF and the anti-inflammation properties of BB, we speculated that BB could alleviate IPF by relieving inflammatory response. Bleomycin (BLM) is an antibiotic used in the treatment of tumors. In clinical situations, the use of BLM was often associated with pulmonary toxicity [20]. BLM-induced IPF is the most commonly used animal model [21]. In our study, the effect and mechanism of BB on IPF were investigated in the BLM-induced mice model. Histopathological changes were determined by routine staining. Pathological indexes and markers of inflammation and fibrosis were selected for analysis. In addition, the tentative mechanism by which BB alleviated IPF was discussed.

## 2. Material and Method

### 2.1. Animals

Male C57BL/6 mice were purchased from Hua Fukang Biological Technology Co., Ltd. (Beijing, China). The animals were maintained under controlled temperature (22 ± 1°C) and humidity (50 ± 5%) laboratory condition, and all mice were acclimatized to the environment for 7 days. Ethical approval from the Animal Experimental Ethics Committee of the Affiliated Hospital of Shandong University of Traditional Chinese Medicine was obtained for using mice (NO. 2021-44).

Mice were arbitrarily allocated into five groups: control, BLM, BLM + 2.5 mg/kg BB, BLM + 5 mg/kg BB, and BLM + 10 mg/kg BB group (12 animals in each group). The BLM group mice were challenged with BLM (2.5 mg/kg) (Dalian Meilun Biotechnology Co., Ltd., Dalian, China) by intratracheal injection to induce IPF. Mice in the sham-operated group were injected intratracheally with equal amounts of sterile saline. After 2 hours, mice in the BB-treated group were daily received different doses of BB (Aladdin regents Co. Ltd., Shanghai, China) by intraperitoneal injection for 21 days. BB was formulated in 20% PEG400 dissolved in saline. Control mice received the equivalent volume of the corresponding vehicle. The mice in each group were anesthetized, and the bronchoalveolar lavage fluid (BALF) was collected. Then, the mice were euthanized, and the lung tissues were obtained, photographed, and weighed.

### 2.2. Morphological Studies

Hematoxylin-eosin (HE) staining was used for general histological changes. The lung samples were fixed in paraformaldehyde, embedded in paraffin, and sectioned. The 5 *μ*m lung slides were stained with hematoxylin (Solarbio Science and Technology, Co., Ltd., Beijing, China) and eosin (Sangon, Shanghai, China).

Masson trichrome staining was used for the detection of IPF. The 5 *μ*m lung slides were stained with Regaud's hematoxylin solution for 6 min. Following rinsed thoroughly with water, sections were dipped in acid fuchsin solution (Sinopharm Chemical Reagent Co., Ltd., Shanghai, China) for 1 min, 1% phosphomolybdic acid solution (Sinopharm Chemical Reagent Co., Ltd., Shanghai, China) for 5 min, and aniline blue solution for 5 min. Then, sections were immersed in 0.2% aqueous glacial acetic acid solution, dehydrated with gradient alcohol, clarified with xylene, and closed with neutral resin. Finally, the tissue sections were observed under a microscope. The severity of alveolitis and fibrosis was scored using the Szapiel scoring system [20].

### 2.3. BALF Cell Count

BALF cells were resuspended in phosphate-buffered saline (PBS) solution, and leukocytes in BALF were quantified by blood cell count. Subsequently, cell smears were fixed in methanol for 15 min and stained with Giemsa staining solution (Nanjing Jiancheng Bioengineering Institute, Nanjing, China). Differential cell counts were analyzed under a light microscope.

### 2.4. Western Blot

Western and IP lysis buffer (Beyotime Biotechnology, Shanghai, China) containing protease inhibitors (Beyotime Biotechnology, Shanghai, China) and nuclear protein extract kit (Beyotime Biotechnology, Shanghai, China) were applied for total and nuclear protein extraction. Protein concentration was measured with bicinchoninic acid (BCA) kit (Beyotime Biotechnology, Shanghai, China). Protein samples resolved by sodium dodecyl sulfate-polyacrylamide gel electrophoresis (SDS-PAGE) (Beyotime Biotechnology, Shanghai, China) were transferred to polyvinylidene fluoride (PVDF) membrane (Millipore, Billerica, MA, USA). After sealing the PVDF membrane for an hour, the membranes were incubated overnight at 4°C with primary antibodies diluted in blocking buffer and secondary antibodies for 45 min at 37°C. Secondary antibodies were detected by enhanced chemiluminescence (ELC) kit (Beyotime Biotechnology, Shanghai, China). Information for primary antibodies was as follows: anti-Fibronectin, anti-NLRP3, anti-ASC (1 : 1000, ABclonal Biotechnology, Wuhan, China), anti-Collagen I (1 : 500), anticleaved caspase-1, anti-JNK, anti-p-JNK, anti-p-p65 (1 : 1000, Affbiotech, Changzhou, China), anti-p65 (1 : 1000, CST, Danvers, MA, USA), anti-*α*-SMA (1 : 1000), histone H3 (1 : 2000), *β*-actin (1 : 10000, Proteintech Group, Inc., Wuhan, China). Information for secondary antibodies was as follows: goat anti-rabbit horseradish peroxidase (HRP) and goat anti-mouse HRP (Beyotime Biotechnology, Shanghai, China).

### 2.5. Reverse Transcription Quantitative Real-Time Polymerase Chain Reaction (RT-qPCR)

Total RNA was extracted using TriPure reagent (BioTeke Bio., Beijing, China). RNA concentration and quality were measured using Nano 2000 (Thermo Fisher Scientific Inc., Pittsburgh, PA, USA). Reverse RNA transcription was performed with BeyoRT II M-MLV reverse transcriptase (Beyotime Biotechnology, Shanghai, China). Synthesized cDNA was subjected to RT-qPCR reaction on Exicycler 96 (Bioneer Corporation, Daejeon, Korea) using SYBR green (Solarbio Science and Technology, Co., Ltd., Beijing, China). Data were quantified using 2^−ΔΔCt^ method and expressed as a ratio of *β*-actin. Information for gene sequence was as follows: MMP-2: forward, CCC CGA TGC TGA TAC TGA; reverse, CTG TCC GCC AAA TAA ACC. MMP-9: forward, TGG GAC CAT CAT AAC ATC AC; reverse, ATG ACA ATG TCC GCT TCG. TIMP-1: forward, TGG GAA ATG CCG CAG ATA; reverse, GCC AGG GAA CCA AGA AGC.

### 2.6. Immunofluorescence (IF) and Immunohistochemistry (IHC) Analysis

IF and IHC were carried out on paraffin-embedded samples after dewaxing and rehydration. For IF staining, the lung sections were incubated with primary antibody against NOD-like receptor family pyrin domain containing 3 (NLRP3) (ABclonal Biotechnology, Wuhan, China) at a dilution of 1 : 100 at 4°C overnight and secondary antibody against Cy3-conjugated goat-anti-rabbit IgG (Invitrogen, Carlsbad, CA, USA) at a dilution of 1 : 200 at room temperature for an hour. 4′,6-diamidino-2-phenylindole (DAPI) (Aladdin regents Co. Ltd., Shanghai, China) was used for for nuclear labeling. The sections were examined under a microscope.

The sections for IHC staining were treated with 3% hydrogen peroxide (Sinopharm Chemical Reagent Co., Ltd., Shanghai, China) for 15 min and blocked by 1% bovine serum albumin (BSA) for 15 min. Then, sections were immunostained with primary antibody (TGF-*β*1, Affbiotech, Changzhou, China) and secondary antibody (HRP-conjugated goat-anti-rabbit IgG, Thermo Fisher Scientific Inc., Pittsburgh, PA, USA). Diaminobenzidine (DAB) (Maxim Biotech, Fuzhou, China) was employed as a developer, and hematoxylin was used for counterstaining. IHC images were captured under a microscope.

### 2.7. Enzyme-Linked Immunosorbent Assay (ELISA)

C-C motif chemokine ligand 2 (CCL2), chemokine C-X-C ligand 10 (CXCL10), tumor necrosis factor (TNF)-*α*, IL-1*β*, and IL-18 levels in BALF were determined using corresponding commercially available ELISA kits. The information of used ELISA kits was as follows: Mouse CCL2 ELISA kit, Mouse CXCL10 ELISA kit, Mouse TNF-*α* ELISA kit, Mouse IL-1*β* ELISA kit, and Mouse IL-18 ELISA kit (MultiSciences Biotech, Hangzhou, China).

### 2.8. Lactate Dehydrogenase (LDH) and Hydroxyproline Content Assay

LDH and hydroxyproline levels in BALF or lung tissues were detected using an LDH assay kit or hydroxyproline assay kit (Jiancheng Bioengineering Institute, Nanjing, China).

### 2.9. Statistical Analysis

The data were analyzed using Graphpad Prism 8.0 (GraphPad Software, La Jolla, CA, USA). All data are presented as mean ± standard deviation (SD). Significance was measured with a one-way analysis of variance (ANOVA) for multiple comparisons. *P*-value less than 0.05 was regarded as indicating statistical significance.

## 3. Results

### 3.1. Bilobalide Attenuated BLM-Induced Pulmonary Fibrosis in Mice

**Figure 1:**
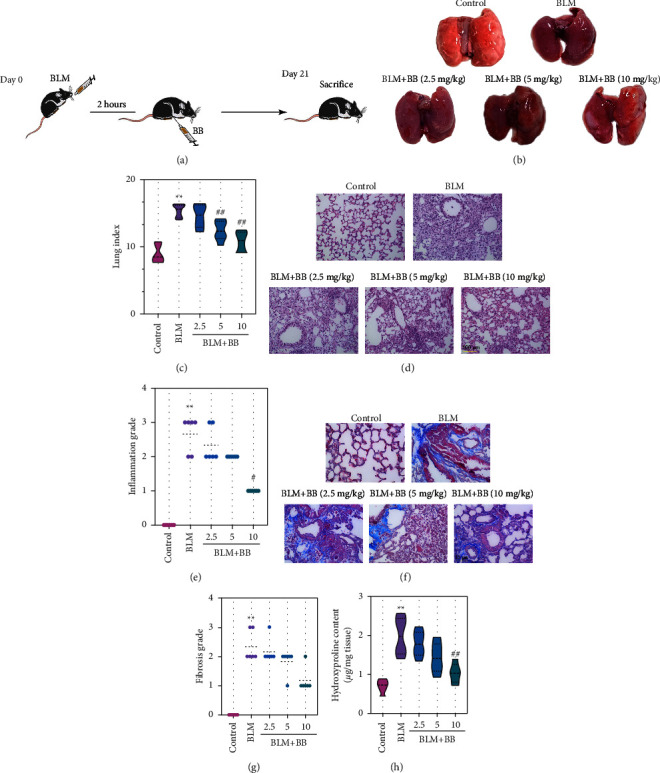
Bilobalide attenuated BLM-induced pulmonary fibrosis in mice. (a) The murine pulmonary fibrosis model was induced with BLM of 2.5 mg/kg, and different doses of bilobalide were administered intraperitoneally after 2 hours. (b) After 21 days, all mice were executed, and the lung samples were excised and photographed. (c) Lung/body weight index. (d) Representative images of H&E lung sections (scale bar = 100 *μ*m). (e) Histological score of H&E staining. (f) Masson trichrome staining of lung sections. Blue color indicates fibrosis (scale bar = 50 *μ*m). (g) Fibrosis score based on Masson staining. (h) Lung hydroxyproline content. Data were reported as means ± SD, *n* = 6. ^*∗∗*^*P* < 0.01 vs. control, ^#^*P* < 0.05 vs. BLM, ^##^*P* < 0.01 vs. BLM. BLM, bleomycin. H&E, hematoxylin and eosin. SD, standard deviation.

In BLM-induced IPF mice, there were signs of structural damage of the lung tissue and ameliorated by BB treatment (Figure 1(b)). Meanwhile, BB treatment obviously attenuated BLM-induced lung index increase (Figure 1(c)). Furthermore, H&E and Masson staining were performed to confirm the alleviation of BB for BLM-induced IPF. H&E staining showed the presence of interstitial septal thickening, inflammatory infiltration, fibrotic nodules, and alveolar structural changes after BLM administration (Figure 1(d)). Masson staining demonstrated the significant collagen fibrosis in the BLM group compared with the control group (Figure 1(f)). In addition, scores of alveolitis and lung fibrosis were significantly higher than that of the control group (Figures 1(e) and 1(g)). Remarkably, these phenomena were alleviated by BB treatment (Figures 1(d)–1(g)). Hydroxyproline content is an important marker reflecting the degree of collagen tissue metabolism and IPF [22]. The high levels of hydroxyproline caused by BLM were significantly reversed by BB administration (Figure 1(h)). Overall, the above results suggested that BB could alleviate BLM-induced IPF.

### 3.2. Bilobalide Down-Regulated the Fibrosis Factor Expression in BLM-Induced Mice

**Figure 2:**
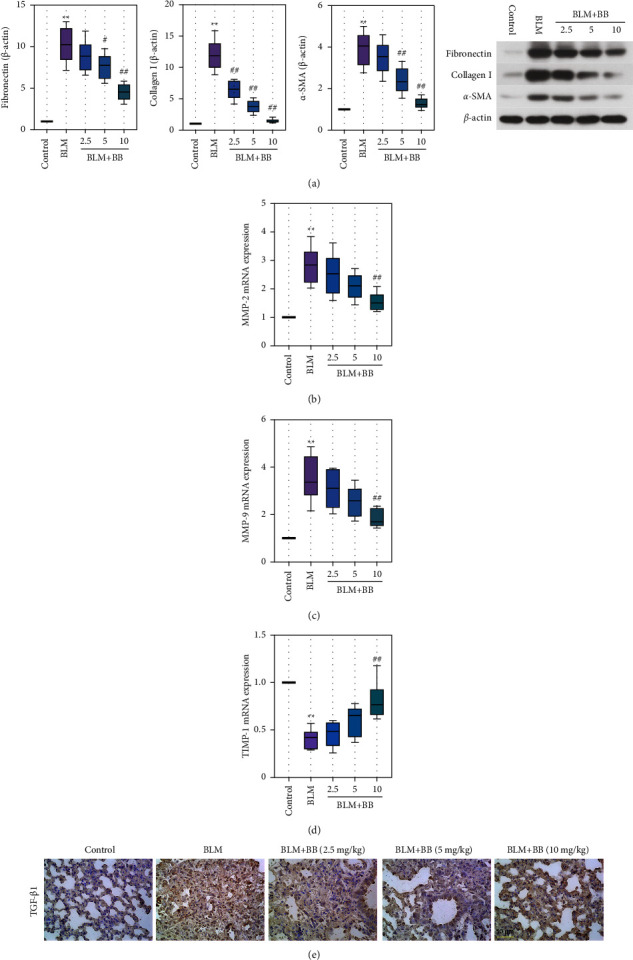
Bilobalide down-regulated the fibrosis factor expression in BLM-induced mice. (a) Analysis of fibronectin, collagen I, and *α*-SMA expression levels was performed by Western blot. (b–d) Gene expression levels of MMP-2 (b), MMP-9 (c), and TIMP-1 (d) were determined via RT-qPCR. (e) TGF-*β*1 immunohistochemical staining (scale bar = 50 *μ*m). Data were reported as means ± SD, *n* = 6. ^*∗∗*^*P* < 0.01 vs. control, ^#^*P* < 0.05 vs. BLM, ^##^*P* < 0.01 vs. BLM. MMP, matrix metallopeptidase. TIMP-1, tissue inhibitor of metalloproteinase 1. RT-qPCR, reverse transcription quantitative real-time polymerase chain reaction.

Subsequently, we measured the protein levels of fibronectin, *α*-SMA and collagen I, biomarkers of fibrosis, with Western blot.  Figure 2(a) shows that those protein levels were enhanced in the BLM group. In contrast, these trends were reversed by BB (Figure 2(a)). At the same time, the gene expression of MMP-2 and MMP-9 was significantly decreased by BB administration and accompanied by increased TIMP-1 expression (Figures 2(b)–2(d)). The expression of pro-fibrotic protein TGF-*β*1 followed the same trend as MMPs (Figure 2(e)).

### 3.3. Bilobalide Mediated Antipulmonary Fibrosis Response by Inhibiting Inflammation

**Figure 3:**
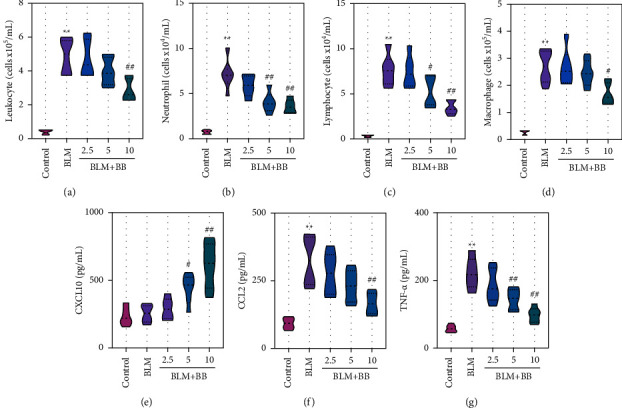
Bilobalide mediated antipulmonary fibrosis response by inhibiting inflammation. Total cell numbers of leukocytes (a), neutrophils (b), lymphocytes (c), and macrophages (d) in BALF were determined by Giemsa staining. CXCL10 (e), CCL2 (f), and TNF-*α* (g) content in BALF. Data were reported as means ± SD, *n* = 6. ^*∗∗*^*P* < 0.01 vs. control, ^#^*P* < 0.05 vs. BLM, ^##^*P* < 0.01 vs. BLM. BALF, bronchoalveolar lavage fluid. CXCL10, chemokine C-X-C ligand 10. CCL2, C-C motif chemokine ligand 2. TNF-*α*, tumor necrosis factor-*α*.

In this model, the anti-inflammation effect of BB was tested. Compared with untreated mice, leukocyte, neutrophil, lymphocyte, and macrophage counts in BALF were significantly increased in BLM-treated mice (Figures 3(a)–3(d)), whereas BB administration markedly decreased these cell counts (Figures 3(a)–3(d)). Meanwhile, BB decreased BLM-induced expression of CCL2 and TNF-*α* (Figures 3(f) and 3(g)). Furthermore, treatment with BB induced a dose-dependent increase in CXCL10 (Figure 3(e)).

### 3.4. Bilobalide Inhibited the Activation of NLRP3 Inflammasome in BLM-Induced Mice

**Figure 4:**
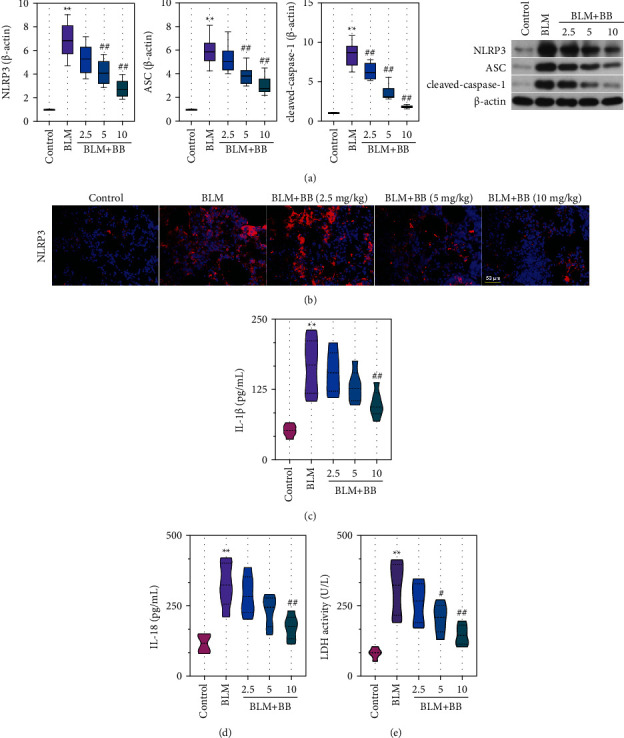
Bilobalide inhibited the activation of NLRP3 inflammasome in BLM-induced mice. (a) Analysis of NLRP3, ASC, and cleaved-caspase-1 expression levels was performed by Western blot. (b) NLRP3 immunofluorescence staining (scale bar = 50 *μ*m). IL-1*β* (c) and IL-18 (d) concentration in BALF. (e) LDH activity in BALF. Data were reported as means ± SD, *n* = 6. ^*∗∗*^*P* < 0.01 vs. control, ^#^*P* < 0.05 vs. BLM, ^##^*P* < 0.01 vs. BLM. NLRP3, NOD-like receptor family pyrin domain containing 3. ASC, apoptosis-associated speck-like protein. IL, interleukin. LDH, lactate dehydrogenase.

To further explore the effect of BB on inflammation, we also examined inflammation-related signal pathways. Data revealed that the protein levels of NLRP3, ASC, and cleaved-caspase-1 in mice exposed to BLM treatment exhibited an obvious elevation (Figure 4(a)). BB therapy remarkably attenuated these protein levels (Figure 4(a)). Indeed, comparable results of NLRP3 were obtained by immunofluorescence (Figure 4(b)). BB treatment significantly decreased the levels of IL-18, IL-1*β*, and LDH in BLM-induced IPF (Figures 4(c)–4(e)).

### 3.5. Bilobalide Suppressed BLM-Induced Inflammation via JNK/NF-*κ*B Pathway

**Figure 5:**
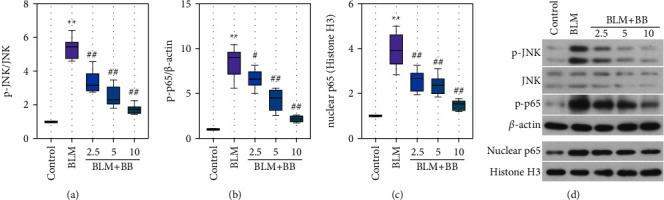
Bilobalide suppressed BLM-induced inflammation via JNK/NF-*κ*B pathway. Analysis of p-JNK (a), p-p65 (b), and nuclear p65 (c). Expression levels were performed by Western blot. (d) Representative Western blot bands for JNK, p-JNK, p-p65, nuclear p65, *β*-actin, and Histone H3. Data were reported as means ± SD, *n* = 6. ^*∗∗*^*P* < 0.01 vs. control, ^#^*P* < 0.05 vs. BLM, ^##^*P* < 0.01 vs. BLM.

A previous study has suggested that JNK/NF-*κ*B might involve in the activation of NLRP3 inflammasome. [23]. As results show, BB significantly inhibited the BLM-induced increase in not only JNK phosphorylation (Figure 5(a)) but also p65 phosphorylation (Figure 5(b)). Meanwhile, the nuclear translocation of p65 was markedly reduced by BB administration (Figure 5(c)).

## 4. Discussion

IPF is a progressive disease with a poor prognosis and limited treatment options. The initial stage of IPF pathogenesis is dominated by alveolar inflammation and massive inflammatory cell infiltration, followed by massive fibroblast proliferation and collagen accumulation in the middle and/or late stages, and finally developed into irreversible interstitial lung fibrosis [24]. Research reported that BLM-induced IPF usually was accompanied by pulmonary oedema resulting in increased lung wet weight [25], which was consistent with our results, while BB could relieve this. BB-induced reduction in lung index showed potential for lung tissue protection. Wet lung weight is used as an indicator of pulmonary inflammation and fibrosis [26]. Our HE and Masson trichrome staining results showed that BLM-treated mice exhibited significant lung histopathological damage as well as an increased degree of inflammatory infiltration and lung fibrosis, indicating successful modeling of IPF. In addition to histopathology, hydroxyproline measurement could also reflect the status of IPF. Hydroxyproline is a unique component of the collagen fibrils in ECM, and one of the main features of IPF is the deposition of ECM [27]. In our results, the degree of alveolar inflammation and fibrosis and hydroxyproline content was remarkably reduced after BB treatment suggesting a significant protective effect of BB on BLM-induced IPF mice.

BLM-induced pulmonary fibrosis is thought to have an early partial inflammatory phase, followed by a fibrotic phase [28]. Enhanced early inflammatory events are critical for the development of pulmonary fibrosis in mice. Inflammatory factors as well as chemokines have been suggested to be key factors in early inflammatory events, during the early stage of pulmonary fibrosis [29]. TNF-*α* was involved in the pro-inflammatory and pro-fibrotic activities of IPF, and its overexpression typically led to the development of IPF [30, 31]. CCL2 belongs to the CC chemokine family and is formed under pathological conditions by pro-inflammatory stimuli such as TNF-*α* [32]. Animal model studies have identified a trend of increased CCL2 in IPF [33, 34]. Our finding showed that BB could reduce BLM-induced lung injury and inflammation, which was supported by reduced cell counts (leukocyte, neutrophil, lymphocyte, and macrophage), the levels of CCL2 and TNF-*α* in BALF, and increased CXCL10 levels. A previous study has shown that the reduction in IPF was associated with a decrease in leukocyte, neutrophil, lymphocyte, and macrophage accumulation [35]. It was noteworthy that in our results, CXCL10 expression showed a gradual increase after BB treatment. CXCL10 is often recognized as an inflammatory chemokine. However, Keane and colleagues [36] found that exogenous CXCL10 inhibits IPF, and its downregulation contributes to IPF lung development. It has antiangiogenic effects and inhibits fibroblast migration in response to the accumulation of fibroblasts in IPF. Tager et al. [37] likewise suggested that CXCL10 functions as an inhibitor of fibroblast chemotactic activity. The changes in up-regulated or down-regulated expression of inflammatory factors and cytokines elucidated the hypothesis of that BB treatment has a mitigating effect on BLM-induced IPF.

Moreover, inflammatory cell infiltration and the release of inflammatory factors are also accompanied by the deposition of ECM, which exacerbates the pathogenesis of IPF. TGF-*β*1 is an important and potent pro-fibrotic mediator in many fibrotic diseases including IPF [38]. It is involved in the development of IPF mainly by stimulating fibroblast activation and modulating EMT [39]. EMT, as an important part of the pathological process of IPF, is mainly manifested by increased expression of the mesenchymal marker *α*-SMA [40]. In addition, the activation of lung fibroblasts is the key rate-limiting step in IPF. Continuous pathogenic stimulation induces fibroblast activation and transforms into myofibroblasts, which secrete large amounts of ECM, leading to ECM deposition and thus accelerating the development of IPF [41]. Collagen I and fibronectin are fibroblast markers [42]. TGF-*β*1 has been proven to regulate the overexpression of fibrogenic proteins, such as Collagen I, fibronectin, and *α*-SMA, and thus exacerbate IPF [43]. Our results showed that BB treatment reduced the expression of Collagen I, fibronectin, *α*-SMA, and TGF-*β*1, indicating that BB might reduce these fibrogenic proteins by inhibiting TGF-*β*1 expression, thereby relieving IPF. Pan et al. [44] have reported that Ginkgo biloba extract EGb761 could decrease the expression of TGF-*β*1 and *α*-SMA to attenuate IPF. MMPs and TIMP-1 are key factors in ECM degradation and remodeling. MMPs promote the development of IPF, and its expression could be regulated by TIMP-1 [45]. The inhibitory or promoting effect of Ginkgo biloba extract-EGb761 on MMPs and TIMP-1 has been shown [46], which is consistent with our results.

Activation of NLRP3 inflammasome plays a crucial role in IPF [47]. Xiong et al. [48] reported that BLM induced a direct fibrogenic effect on IPF by up-regulating collagen expression and promoting inflammatory factors release via JNK/NF-*κ*B pathway. However, the relationship between BB and NLRP3 or JNK/NF-*κ*B signaling pathways in IPF has not been clarified. The results of this study showed that BB significantly reduced BLM-induced activation of NLRP3 and NF-*κ*B. In addition, BLM increased the phosphorylation of JNK, while BB treatment reversed this. The results showed that BB was involved in the protective effect of lung fibrosis mice through NLRP3 and JNK/NF-*κ*B signaling pathways. This result was supported by previous studies. The report showed that high levels of JNK phosphorylation were closely associated with fibroblast to integrate fibroblast differentiation in IPF [49]. By targeting downstream signaling molecules such as NF-*κ*B, JNK exacerbated BLM-induced lung tissue fibrosis and inflammation [50]. NF-*κ*B is known to be upstream of NLRP3 [51], and the overexpression of NLRP3 exacerbates the release of inflammatory factors IL-1*β* and IL-18. The report has shown that Ginkgo biloba extract EGb761 had the potential to inhibit the activation of the NF-*κ*B pathway [44]. In this study, we reported for the first time that BB inhibited the activation of JNK/NF-*κ*B and NLRP3 in IPF, but we have only preliminarily explored the mechanisms. Endeavors to dissect the molecular mechanisms of JNK, NF-*κ*B, and NLRP3 signaling will provide more valid and reliable evidence for the role of BB in protecting against BLM-induced pulmonary inflammation and fibrosis.

## 5. Conclusions

**Figure 6:**
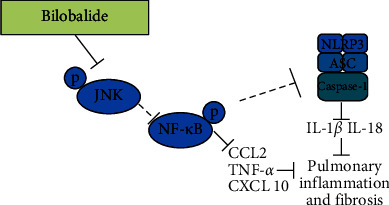
Diagram depicting the regulation mechanism of bilobalide in ameliorating pulmonary fibrosis. Bilobalide inhibited inflammatory cytokines release by reducing the phosphorylation of the JNK/NF-*κ*B pathway and the activation of NLRP3 to inhibit the inflammatory response in pulmonary fibrosis.

In conclusion, we investigated the effects of BB on BLM-induced pulmonary fibrosis and elucidated the underlying mechanisms. Administered in the early inflammatory phase, BB had a protective effect on pulmonary fibrosis in mice induced by BLM, which might be related to the inhibition of early inflammatory response and amelioration of ECM deposition. The underlying mechanism might be via the inhibition of the JNK/NF-*κ*B/NLRP3 signal pathway. A summary of the proposed mechanism is shown in Figure 6. Overall, this study may provide some new insights into the mechanism of action of BB in the treatment of pulmonary inflammation and fibrosis.

## Data Availability

The data used to support the findings of this study are available from the corresponding author upon reasonable request.
